# Mapping the evolution and research landscape of Retzius-sparing robot-assisted radical prostatectomy: a bibliometric analysis

**DOI:** 10.1007/s11701-026-03400-x

**Published:** 2026-04-11

**Authors:** Çağrı Öktem, Ali Kaan Yıldız, Yusuf Gökkurt, Arif Bedirhan Bayraktar, Ahmet Varan, Ömer Furkan Erbay

**Affiliations:** 1 Department of Urology, Kulu State Hospital, Konya, Turkey; 2 Department of Urology, Ankara Bilkent City Hospital, Ankara, Turkey; 3 Department of Urology, Yozgat City Hospital, Yozgat, Turkey; 4 Department of Urology, Haymana State Hospital, Ankara, Turkey

**Keywords:** Retzius-sparing radical prostatectomy, Robot-assisted radical prostatectomy, Urinary continence, Bibliometrics, Prostate cancer

## Abstract

This study aimed to analyze the scientific literature on Retzius-sparing robot-assisted radical prostatectomy (RS-RARP) using bibliometric methods to identify temporal trends in publication output, major research themes, and the structural characteristics of the field. Original research articles published in English between 2011 and 2025, indexed in the Web of Science Core Collection database and included in the Science Citation Index Expanded (SCI-E), were analyzed. Publications retrieved through a predefined search strategy were evaluated in terms of annual scientific production, citation indicators, journal distribution, highly influential studies, and author and keyword networks. Bibliometric analyses and visualizations were performed using Biblioshiny (Bibliometrix R package) and VOSviewer. A total of 93 original research articles met the inclusion criteria. Annual publication output increased markedly after 2017, reaching a peak in 2023. Journal of Robotic Surgery emerged as the most productive journal (*n* = 12), whereas European Urology, Journal of Urology, and BJU International demonstrated the highest impact and visibility. Italy (*n* = 24), the United States (*n* = 21), and China (*n* = 17) were identified as the leading contributors to scientific output. Citation analyses showed that the intellectual foundation of the field is centered on a limited number of seminal studies. Keyword and network analyses revealed a predominant focus on functional outcomes, refinement of surgical techniques, and learning curve–related themes. Overall, the RS-RARP literature represents an increasingly mature and expanding research domain, and these findings provide a quantitative overview of its current structure while offering guidance for future clinical and methodological investigations.

**Supplementary Information:**

The online version contains supplementary material available at 10.1007/s11701-026-03400-x.

## Introduction

Prostate cancer is the second most frequently diagnosed cancer among men. In 2020, approximately 1.4 million new cases and 375,000 deaths were reported worldwide [1]. Radical prostatectomy represents one of the cornerstones of curative treatment for prostate cancer, and surgical success should be evaluated not only in terms of oncological control but also with respect to postoperative functional outcomes [2]. With the widespread adoption of robot-assisted radical prostatectomy, surgical techniques have evolved toward enhancing anatomical preservation and enabling a more rapid recovery of patients’ quality of life [2, 3].

Among these technical advancements, the Retzius-sparing (posterior) approach has been defined as a distinct surgical concept aimed at preserving the Retzius space and the anterior supportive structures [3]. Although the literature includes clinical studies, randomized controlled trials, systematic reviews, and meta-analyses evaluating the functional and oncological outcomes of this approach, the existing body of evidence has largely focused on patient outcomes. Notably, there is a lack of bibliometric studies that quantitatively assess the dynamics of scientific production, research trends, and the intellectual structure of the field [4–6].

The aim of this study is to analyze the literature on Retzius-sparing robot-assisted radical prostatectomy using bibliometric methods, with the objective of elucidating the temporal evolution of scientific output, identifying prominent research themes, and characterizing the structural features of the literature. By addressing this gap, the study seeks to provide a guiding framework for future clinical and methodological research.

## Materials and methods

### Search strategy and data collection

This bibliometric study was conducted using the Web of Science (WoS) Core Collection database, and publications from 2011 to 2025 were retrieved using the “topic” (TS) search field. The search strategy was constructed as follows: “TS = (“Retzius-sparing” OR “Retzius sparing” OR “Retzius-preserving” OR “Retzius preserving” OR “Retzius-sparing radical prostatectomy” OR “Retzius sparing radical prostatectomy” OR “Retzius-sparing robotic prostatectomy” OR “Retzius sparing robotic prostatectomy” OR “Retzius-sparing robot-assisted radical prostatectomy” OR “Retzius sparing robot assisted radical prostatectomy”)”. Data retrieval was performed on January 28, 2026.

The inclusion criteria were defined as publications indexed in WoS and published in journals included in the Science Citation Index Expanded (SCI-E), written in English, classified as original research articles, and retrieved using the predefined search strategy. Articles directly related to the topic were manually selected. Publication types other than original research, including case reports, case series, narrative reviews, systematic reviews, and meta-analyses, were excluded from the study. Data collection and manual screening of the records were performed independently by two authors (Ç.Ö. and Y.G.). Discrepancies between reviewers were resolved through discussion and consensus, and when necessary, a third author (A.K.Y.) was consulted to reach a final decision.

**Fig. 1:**
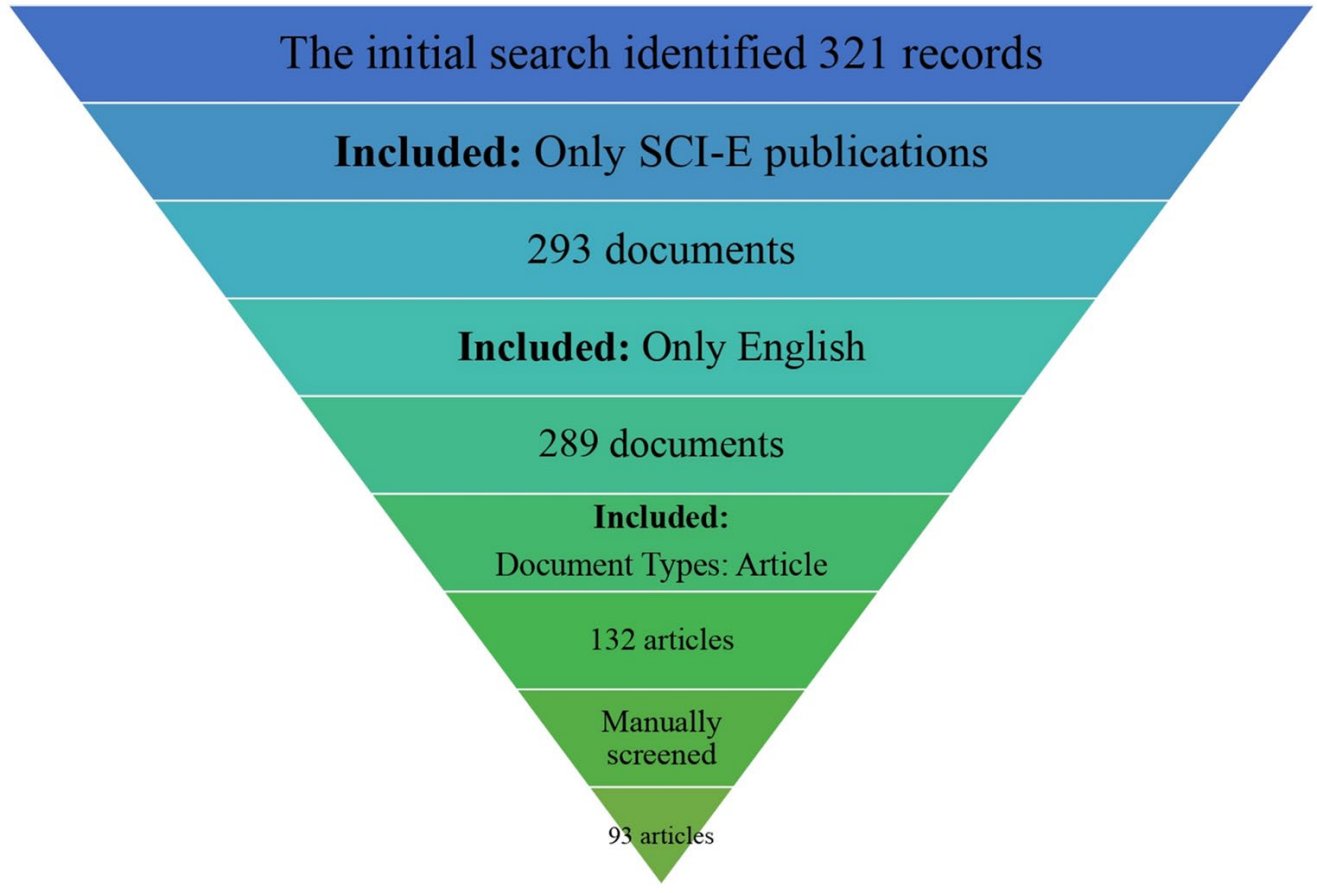
Flow diagram of the study selection process

A total of 321 publications were initially identified. After applying the inclusion and exclusion criteria, 132 articles remained. Manual screening for topic relevance yielded a final dataset of 93 original research articles published between 2011 and 2025 (Fig. 1).

### Data analysis tools

Microsoft Excel (version 2016) was used for data organization, analysis, and visualization [7]. Bibliometric analyses were performed using Biblioshiny (version 4.1.3), the web-based interface of the Bibliometrix R package, while network and visual analyses were conducted using VOSviewer software (version 1.6.20) [8–10].

Data downloaded from WoS were imported into the Biblioshiny environment, and duplicate records and missing information were checked and corrected prior to analysis. Temporal trends in scientific production and citation dynamics were analyzed using annual publication counts (Annual Scientific Production) and average citations per year (Average Citation Per Year). Source structure and journal productivity were evaluated using analyses of the most relevant sources and sources’ production over time. The intellectual foundation of the literature was examined using the most globally cited documents and Reference Publication Year Spectroscopy (RPYS).

To illustrate the structural patterns of scientific knowledge production and dissemination by revealing relationships among influential publications, authors, and affiliated institutions, a three-field plot analysis was performed. The development of the conceptual structure and thematic concentrations was assessed using thematic map analysis. Geographic contributions and collaboration patterns were evaluated using country collaboration maps and corresponding author country analyses.

Network and relationship analyses were conducted using VOSviewer. Co-authorship and keyword co-occurrence analyses were performed. The full counting method and association strength normalization were applied throughout. In network visualizations, units were defined as nodes and relationships between units as links. Total Link Strength (TLS) was used to represent overall network connectivity.

In the co-authorship analysis, network and overlay visualizations were generated for authors. Node size reflected the authors’ relative weight within the network, links represented collaboration strength, and the overlay visualization was based on average publication year (APY). The minimum thresholds for inclusion were set at one document and zero citations.

A country-level co-authorship network visualization was also generated. Node size reflected country weight, and links represented international collaboration strength. The minimum thresholds for inclusion were set at one document and zero citations.

Relationships among keywords were examined using co-occurrence analysis to reveal the conceptual structure of the research field, based on author-provided keywords. In the network visualization, each keyword was represented as a node, with links indicating their co-occurrence within the same publications. Node size reflected the frequency of keyword usage, and the minimum inclusion threshold was set to one occurrence.

### Data cleaning

Prior to data analysis, a comprehensive data-cleaning process was applied. To enhance consistency in VOSviewer analyses, a thesaurus file was used to merge different spellings of author names, country names, and keywords (Supplementary Materials 1, 2, and 3). These procedures were implemented to improve the reliability of the bibliometric analyses and to enable more accurate interpretation of the resulting visualizations.

## Results

**Fig. 2:**
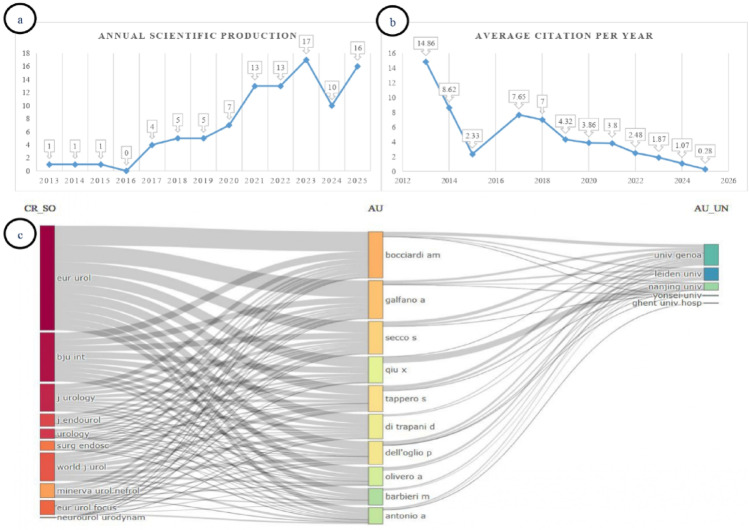
(**a**) Annual scientific production, (**b**) Average citations per year, (**c**) Three-field plot showing relationships between journals, authors, and institutions

Annual scientific production remained at one publication per year between 2013 and 2015, with no publications recorded in 2016 (Fig. 2a). The number of publications increased after 2017, reaching a peak annual output of 17 publications in 2023. In 2024, the number declined to 10 publications, followed by a renewed increase to 16 publications in 2025.

The highest average citation per year was observed in 2013 (14.86), whereas the lowest value was recorded in 2025 (0.28) (Fig. 2b).

In the three-field plot analysis, European Urology was identified as the source with the highest flow value (559), followed by BJU International (261), World Journal of Urology (150), and Journal of Urology (146) (Fig. 2c). At the author level, Bocciardi AM and Galfano A emerged as the researchers with the strongest collaborative connections. Institutional analysis demonstrated that publications were predominantly affiliated with the University of Genoa, followed by Leiden University and Nanjing University.

**Fig. 3:**
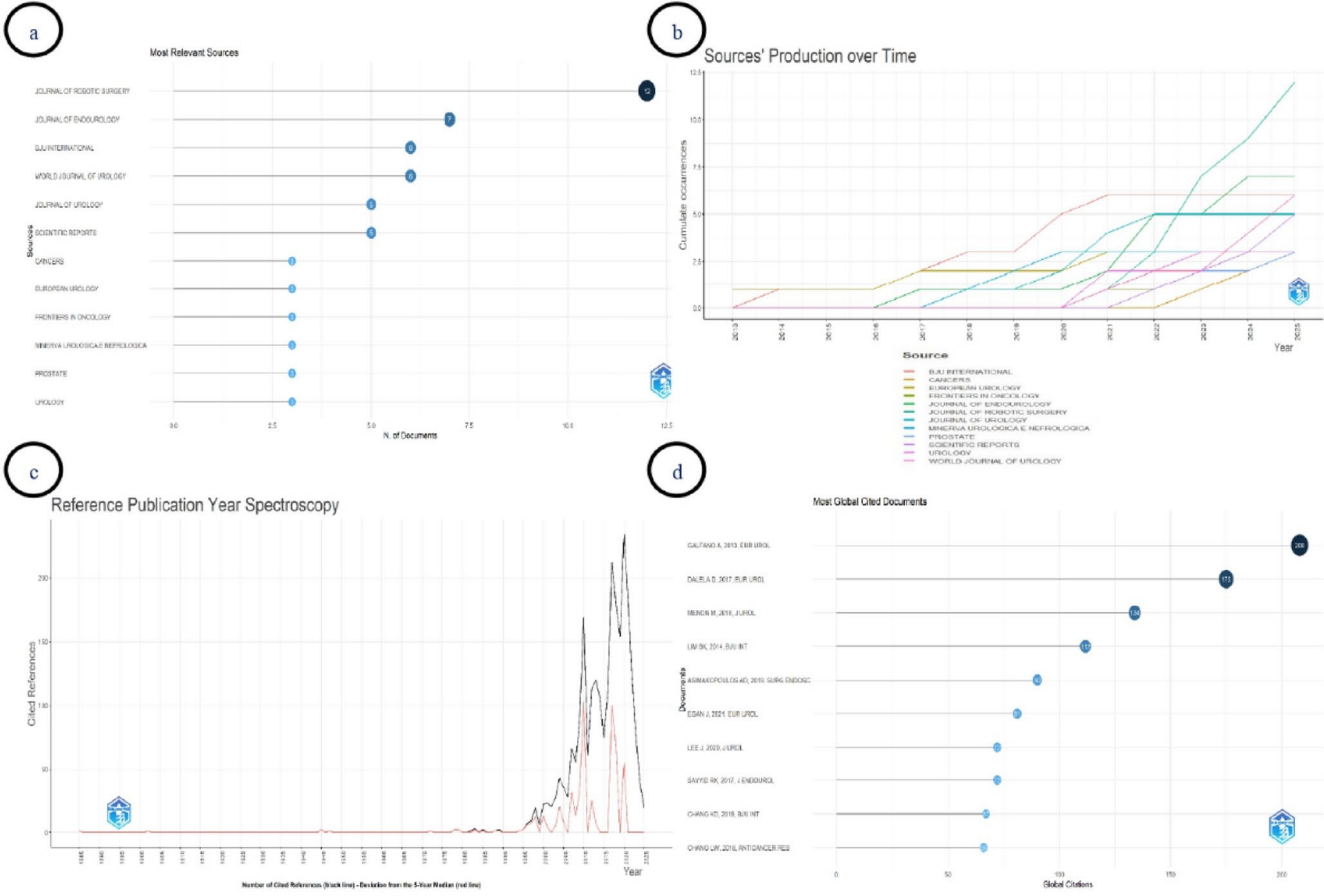
(**a**) Most relevant sources by number of publications, (**b**) Sources’ production over time, (**c**) Reference Publication Year Spectroscopy, (**d**) Top 10 most globally cited documents

When the most relevant sources and temporal publication output were evaluated together, publications were found to be concentrated in specific journals, with this concentration increasing over time (Fig. 3a and b). Overall, the highest number of publications was recorded in Journal of Robotic Surgery, which ranked first with 12 articles and became particularly prominent in annual output by 2025 following a rapid increase after 2021. This was followed by Journal of Endourology (*n* = 7), BJU International, and World Journal of Urology (each *n* = 6). During the early period (2013–2016), publications were limited in number and were mainly published in European Urology and BJU International. After 2017, a gradual increase was observed, and after 2020 both publication volume and journal diversity expanded markedly. Journal of Urology and Scientific Reports made moderate contributions, whereas several other journals contributed smaller numbers of publications.

RPYS showed a marked increase in cited references from the late 1990s onward, with the most prominent clustering observed between 2010 and 2021 (Fig. 3c).

The top 10 most globally cited articles are shown in Fig. 3d [2–4, 11–17]. The most cited study was published by Galfano et al. in 2013 in European Urology (208 citations), followed by Dalela et al. (2017, 175 citations) and Menon et al. (2018, 134 citations) [2–4]. Overall, highly cited publications were predominantly published in leading journals such as European Urology, Journal of Urology, and BJU International.

**Fig. 4:**
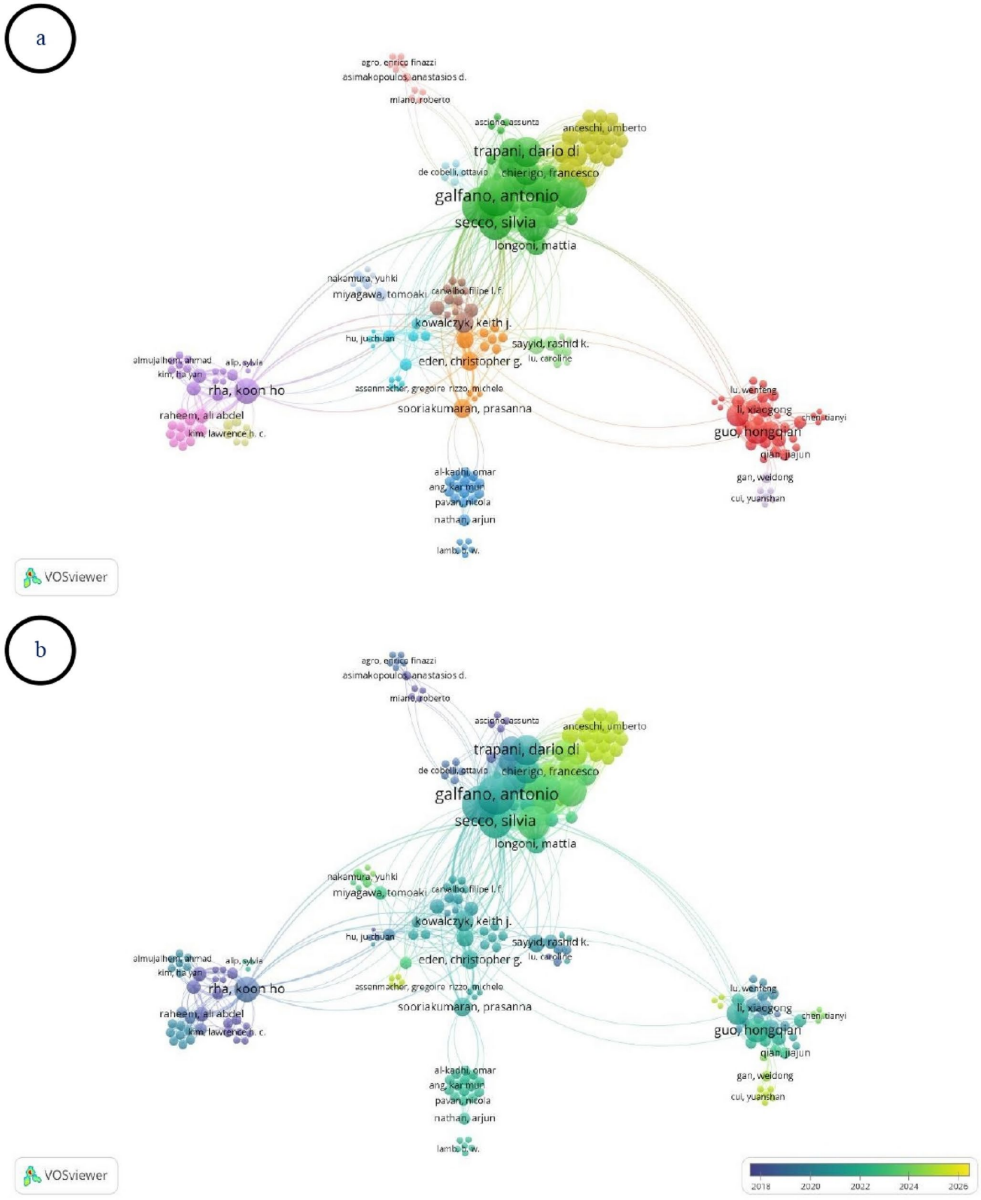
(**a**) Co-authorship network visualization among authors, (**b**) Overlay visualization of the co-authorship network based on average publication year

The co-authorship network is shown in Fig. 4a, with temporal patterns presented in Fig. 4b. A total of 481 authors were identified, of whom 219 interconnected authors were included in the co-authorship analysis. The network comprised 15 clusters and 1,607 links (TLS = 2,386). Antonio Galfano ranked first across all indicators (18 documents, 464 citations, TLS = 208). The second and third positions in terms of document count and TLS were shared by Aldo Massimo Bocciardi (17 documents, TLS = 184) and Silvia Secco (14 documents, TLS = 157). Overlay visualization demonstrated that publications by authors with high TLS values were predominantly concentrated in the post-2019 period.

**Fig. 5:**
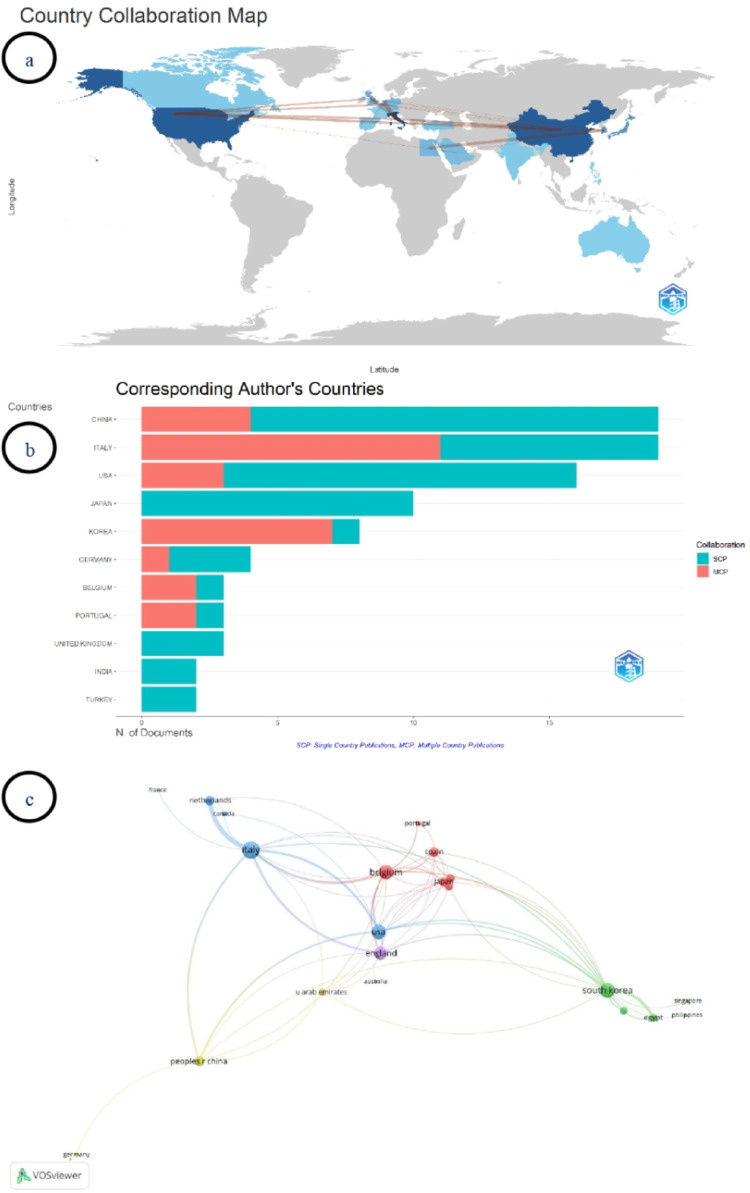
(**a**) International country collaboration map, (**b**) Corresponding authors’ countries showing single-country publications (SCP) and multiple-country publications (MCP), (**c**) Country co-authorship network

World map visualization indicated that publications were mainly concentrated in East Asia, Europe, and North America (Fig. 5a). Italy, the United States (US), and China emerged as the countries with the highest publication intensity. More than 60 bilateral country links were identified. The strongest collaboration was between Italy and the Netherlands (*n* = 8), followed by South Korea–Egypt (*n* = 5).

In the corresponding author country analysis, China and Italy ranked first with the highest number of publications (19 documents each) (Fig. 5b). The US ranked third with 16 documents, followed by Japan with 10 documents. Single-country publications (SCP) were predominant in Japan, the United Kingdom, India, and Turkey, whereas the proportion of multiple-country publications (MCP) was higher in countries such as South Korea (87.5%), Italy (57.9%), and Belgium (66.7%).

The country-level collaboration network is presented in Fig. 5c. Of the 23 countries identified, 22 interconnected countries were included in the co-authorship analysis. The network consisted of five clusters, 64 links, and a TLS of 103. The countries with the highest number of publications were Italy (24), the US (21), China (17), Japan (11), and South Korea (10). The highest TLS values were observed for Italy (32), the US (23), and South Korea (23), while the highest citation counts were recorded for the US (687), Italy (683), and South Korea (417).

**Fig. 6:**
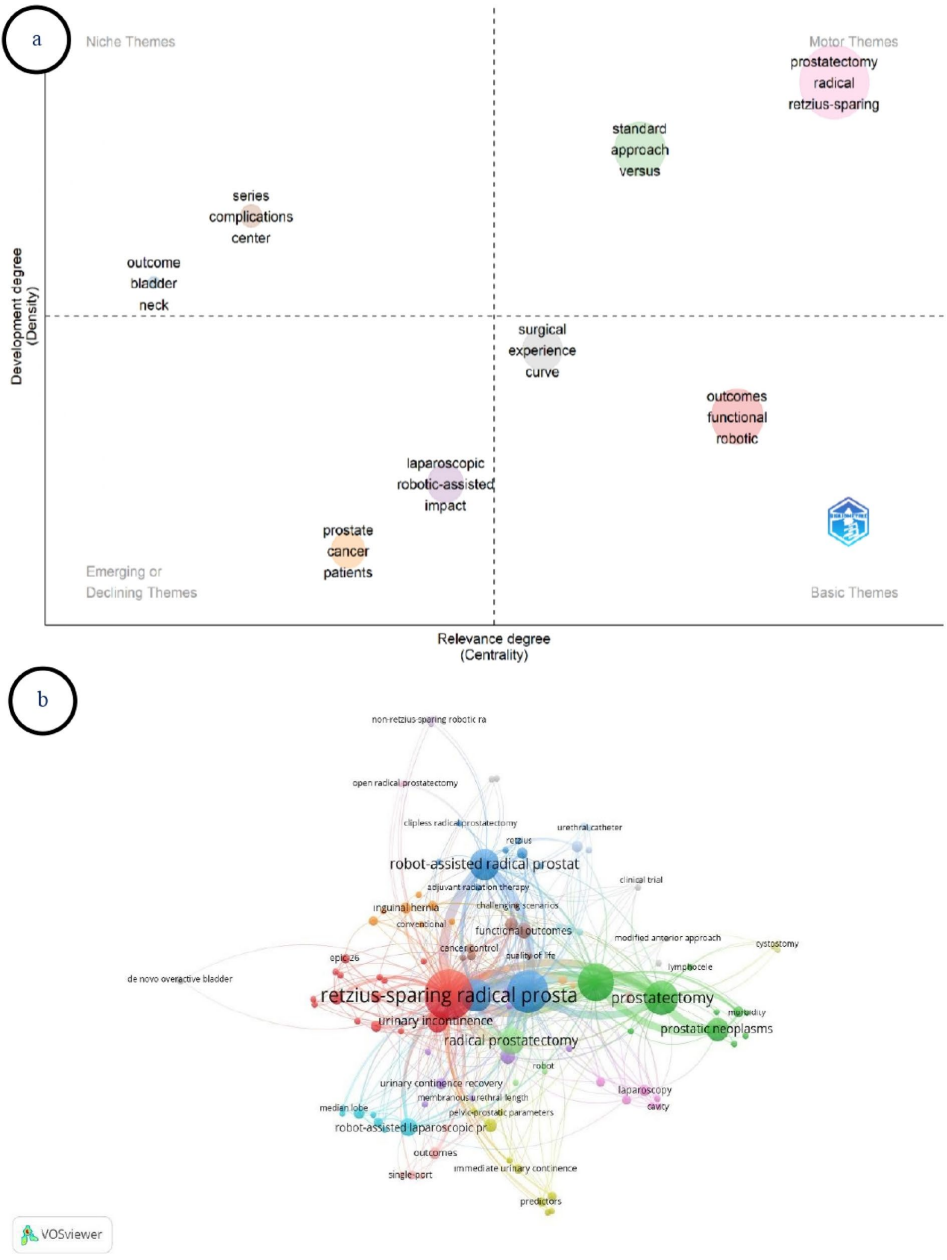
(**a**) Thematic map of research themes, (**b**) Keyword co-occurrence network based on authors’ keywords

In the thematic map analysis, the keywords *prostatectomy*, *radical*, and *retzius-sparing* were located in the motor themes quadrant, exhibiting high centrality and high density (Fig. 6a). The themes *standard*, *approach*, and *versus* were also positioned within the motor themes. The keywords *outcomes*, *functional*, and *robotic* were located in the basic themes area, whereas *surgical*, *experience*, and *curve* showed high centrality but lower density, placing them in the central–lower quadrant. The themes *outcome*, *bladder*, and *neck* were classified as niche themes. The keywords *prostate*, *cancer*, and *patients* were positioned in the emerging or declining themes quadrant, characterized by low centrality and low density.

Keyword co-occurrence analysis identified 117 interconnected keywords arranged in 24 clusters (TLS = 814) (Fig. 6b). The most frequently co-occurring keywords with the highest TLS values were *retzius-sparing radical prostatectomy* (57 occurrences, TLS = 226), *prostate cancer* (40 occurrences, TLS = 172), *robotic surgery* (31 occurrences, TLS = 120), *prostatectomy* (27 occurrences, TLS = 101), *urinary continence* (23 occurrences, TLS = 89).

## Discussion

This bibliometric study provides a comprehensive evaluation of scientific production trends, research focuses, and the structure of international contributions in original studies published between 2011 and 2025 in the field of Retzius-sparing radical prostatectomy. The findings indicate that this surgical approach has attracted increasing attention, particularly over the past decade, and that the literature has progressively evolved toward a focus on functional outcomes and patient-centered results.

Annual scientific production data demonstrate a marked increase in publication volume after 2017. This growth may be attributed to the broader adoption of robotic surgery in clinical practice and to an expanded definition of surgical success that extends beyond oncological control to include early continence and quality of life outcomes. The higher average citation rates observed in early publications suggest that these studies established the conceptual foundation of the field and became reference points within the literature. In contrast, the relatively lower citation levels of more recent publications may be explained by their shorter time since publication and the limited opportunity for citation accumulation, reflecting citation window bias rather than necessarily lower scientific relevance. In addition, citation trends were evaluated using average citations per year (citation density), as illustrated in Fig. 2b, allowing for a more balanced comparison of citation impact across different publication years.

Evaluation of the intellectual structure of the literature reveals that research activity has been concentrated around specific centers and specialized journals. Conceptually, studies have primarily focused on optimization of surgical technique, the learning curve, and improvement of functional outcomes. This trend is consistent with earlier reports demonstrating that surgical experience and technical refinement have played a central role in shaping the radical prostatectomy literature since the early laparoscopic era [18]. These themes have also influenced clinical practice, as increasing surgical experience and continued technical refinement have contributed to improved functional outcomes and earlier continence recovery following RS-RARP [3, 4, 12]. In addition, insights into the learning curve have supported safer adoption of the technique.

Journal-based evaluations indicate that the Journal of Robotic Surgery has emerged as the most prolific journal in the Retzius-sparing radical prostatectomy literature, thereby gaining notable visibility within the field. By providing extensive coverage of studies focusing on surgical details and technical feasibility, this journal has contributed substantially to the technical depth of the literature [19, 20]. In addition, Journal of Endourology and World Journal of Urology have supported the diversification of the field through publications reporting clinical outcomes across different surgical practices and patient subgroups [21, 22]. European Urology and BJU International, on the other hand, have played a key role in the initial description of the technique and the reporting of early clinical outcomes, thereby enhancing its international recognition [13, 16].

Patterns of collaboration among authors and institutions demonstrate that the Retzius-sparing approach initially developed within a limited number of centers and subsequently disseminated through strong international collaborations. This dissemination has facilitated evaluation of the technique across diverse patient populations and surgical platforms. Approaches aimed at preservation or reconstruction of the Retzius space have received increasing attention, particularly with respect to achieving early continence [23].

Country-level analyses identify Italy, the United States, and China as the leading contributors to scientific output in this field. Italy has played a pioneering role in the development of the technique and in the systematic reporting of both functional and oncological outcomes during the early period [3, 12, 24]. Studies originating from the United States have largely focused on evaluating the impact of surgical care pathways and robotic platform variations on clinical safety and early outcomes [25, 26]. In contrast, China-based studies have emphasized the role of magnetic resonance imaging–based anatomical parameters in predicting functional and oncological outcomes following the Retzius-sparing approach [27, 28]. These findings are thought to provide clinically meaningful insights, particularly in guiding patient selection, perioperative planning, and technique adaptation across diverse surgical settings.

Thematic analyses indicate that research topics have increasingly concentrated on early continence, functional outcomes, and optimization of minimally invasive surgery. Evidence suggesting that the Retzius-sparing approach offers superior early continence outcomes compared with standard robot-assisted radical prostatectomy has been supported by both single-center studies and large-scale meta-analyses [2, 29, 30].

These findings may also be interpreted in light of contemporary guideline evolution, suggesting that current research trends are broadly aligned with the increasing emphasis on functional outcomes and quality of life in clinical decision-making; accordingly, the growing focus on early continence and techniques such as RS-RARP in the literature appears consistent with this shift [1, 31].

Several limitations of this study should be acknowledged. The Web of Science Core Collection was selected due to its high-quality indexing standards and comprehensive citation data, which are essential for reliable bibliometric analysis. However, the exclusive use of this database may have resulted in the exclusion of relevant studies indexed in other databases such as Scopus or PubMed, particularly in a rapidly evolving surgical field. Additionally, restricting the analysis to English-language publications may have led to the omission of some regional studies. Bibliometric indicators also do not directly reflect methodological quality or clinical heterogeneity among studies. In addition, citation counts may be influenced by publication time and research visibility, and therefore should be interpreted with caution.

Nevertheless, a major strength of this study lies in its multidimensional evaluation of the Retzius-sparing radical prostatectomy literature, extending beyond quantitative productivity metrics to encompass research trends, journal focus, and global contribution patterns. This approach may reflect a shift in the field from a technically driven innovation toward a research paradigm increasingly focused on patient-oriented clinical outcomes.

In conclusion, the literature on Retzius-sparing radical prostatectomy appears to be evolving into a more mature structure, characterized by growing scientific output, expanding international collaboration, and an increasing emphasis on patient-centered research priorities. Future bibliometric analyses integrating multiple databases may provide a more comprehensive mapping of the field. Studies in this field may benefit from not only targeting surgical and technological optimization but also strengthening existing findings through multicenter designs and long-term follow-up.

## Supplementary Information

Below is the link to the electronic supplementary material.

Supplementary Material 1

Supplementary Material 2

Supplementary Material 3

Supplementary Material 4

## Data Availability

The datasets generated and/or analyzed during the current study are available in Supplementary Material 4.
